# Cagelike Octacopper Methylsilsesquioxanes: Self-Assembly in the Focus of Alkaline Metal Ion Influence—Synthesis, Structure, and Catalytic Activity

**DOI:** 10.3390/molecules28031211

**Published:** 2023-01-26

**Authors:** Alexey N. Bilyachenko, Ivan S. Arteev, Victor N. Khrustalev, Anna Y. Zueva, Lidia S. Shul’pina, Elena S. Shubina, Nikolay S. Ikonnikov, Georgiy B. Shul’pin

**Affiliations:** 1 A. N. Nesmeyanov Institute of Organoelement Compounds, Russian Academy of Sciences, Vavilov Str. 28, 119991 Moscow, Russia; 2 Higher Chemical College, Mendeleev University of Chemical Technology of Russia, Miusskaya Sq. 9, 125047 Moscow, Russia; 3 Research Institute of Chemistry, Peoples’ Friendship University of Russia (RUDN University), Miklukho-Maklay Str. 6, 117198 Moscow, Russia; 4 Zelinsky Institute of Organic Chemistry, Russian Academy of Sciences (RAS), Leninsky Prospect 47, 119991 Moscow, Russia; 5 Semenov Federal Research Center for Chemical Physics, Russian Academy of Sciences, Ulitsa Kosygina 4, 119991 Moscow, Russia; 6 Chair of Chemistry and Physics, Plekhanov Russian University of Economics, Stremyannyi Pereulok, Dom 36, 117997 Moscow, Russia

**Keywords:** metallasilsesquioxanes, cage-like compounds, molecular recognition, oxidative catalysis, alkanes, alkyl hydroperoxide

## Abstract

A family of unusual octacopper cage methylsilsesquioxanes **1**–**4** were prepared and characterized. Features of their cagelike (prismatic) structure were established using X-ray diffraction studies. Effects of distortion of prismatic cages **1**–**4** due to variation of (i) additional alkaline metal ions (K, Rb, or Cs), (ii) combination of solvating ligands, and (iii) nature of encapsulating species were found. Opportunities for the design of supramolecular 1D extended structures were found. These opportunities are based on (i) formate linkers between copper centers (in the case of Cu_8_K_2_-based compound **2**) or (ii) crown ether-like contacts between cesium ions and siloxane cycles (in the case of Cu_8_Cs_2_-based compound **4**). Cu_8_Cs_2_-complex **4** was evaluated in the catalysis of alkanes and alcohols. Complex **4** exhibits high catalytic activity. The yield of cyclohexane oxidation products is 35%. The presence of nitric acid is necessary as a co-catalyst. The oxidation of alcohols with the participation of complex **4** as a catalyst and tert-butyl hydroperoxide as an oxidizer also proceeds in high yields of up to 98%.

## 1. Introduction

Cagelike metallasilsesquioxanes (CLMSs) are a wide family of polynuclear metallacomplexes, providing a kaleidoscopic variety of molecular architectures [1,2,3,4,5,6,7,8,9,10,11] as well as impressive diversity of further applications. The latter comprises approaches to molecular magnets (including behaviors of single-molecule magnets or spin glass types) [12,13,14,15,16,17,18] and objects with photophysical properties (including the first example of a lanthanide-based luminescent thermometer) [19,20,21,22]. In the context of material science, CLMSs were successfully applied for the versatile design of functional MOFs and coordination polymers [23,24,25,26], anodes [27,28], biomedical agents [29], and fire retardants [30,31,32,33,34,35]. Numerous reviews summarize the effectiveness of CLMS in various catalyzed reactions, including oxidative C–H bond activations [36,37,38,39,40,41,42]. Among recent results we could mention activity in CO_2_ cycloaddition [43], Chan-Evans-Lam coupling [44], biomass transformations [45], and oxidative amidation [46]. Considering the significant potential of copper-based CLMSs as catalysts [47,48,49,50], we were interested in designing new types of these compounds. Numerous results pointed out the significant influence of the nature of the substituent at the silicon center on molecular geometry of arising CLMSs, e.g., for nickel-based (phenyl- [51,52,53,54] vs. 4-vinylbenzyl-substituted [55] CLMSs), titanium-based (*t*-Bu vs. (2,6-*i*Pr_2_C_6_H_3_)N(SiMe_3_)-substituted [56,57] CLMSs), aluminum-based (cyclopentyl [58] vs. cyclohexyl-substituted [59] CLMSs) compounds. In turn, it is well known that the archetype of phenyl-substituted copper CLMSs is a hexanuclear prismatic structure [1,6,8,11,48,49]. Our newest results describing methyl-substituted Cu-CLMSs’ synthesis, structure determination, and evaluation in catalysis are presented below.

## 2. Results

### 2.1. Synthesis and Structure

**Scheme 1:**
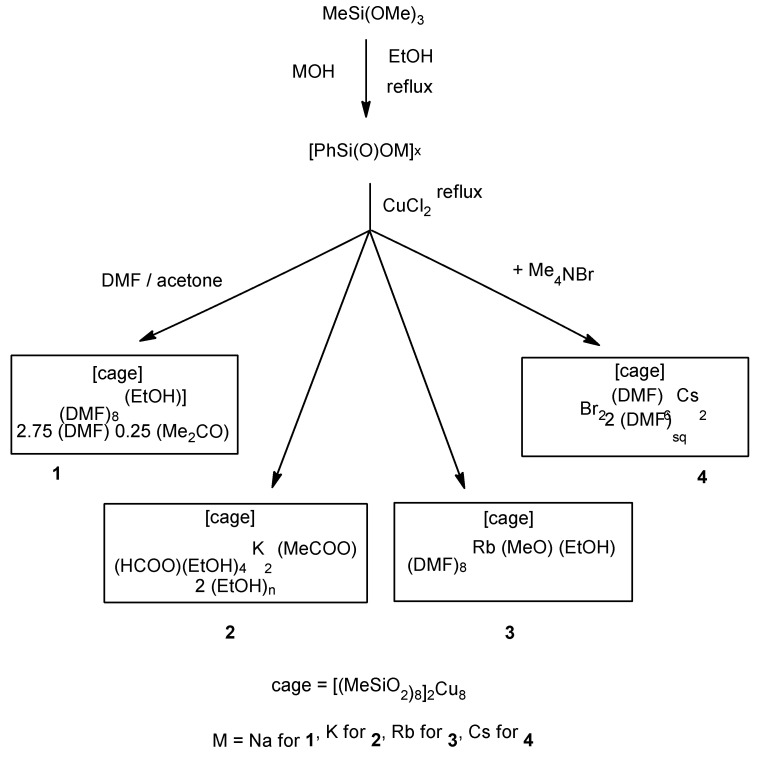
General scheme of synthesis of cagelike octacopper methylsilsesquioxanes **1**–**4**.

**Figure 1:**
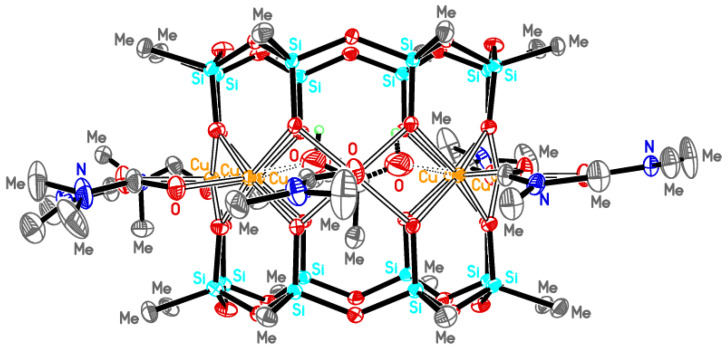

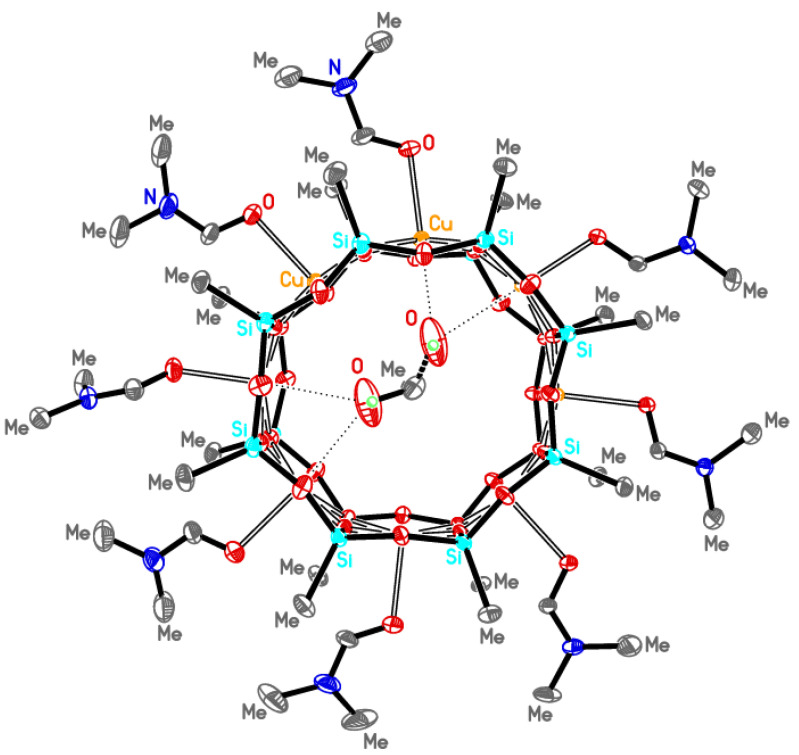
Two projections of molecular structure of complex [(MeSiO_2_)_8_]_2_Cu_8_(DMF)_8_ (EtOH)] • 2.75 (DMF) • 0.25 (Me_2_CO) **1**; the solvate dimethylformamide and acetone molecules are not shown. Hydrogen atoms are presented as green dots (the same concerns Figure 2, Figure 3, Figure 4, Figure 5 and Figure 6).

It is known that variations of the nature of alkaline metal ions strongly influence the structural features of phenylsurrounded CLMSs, especially in the context of supramolecular aggregation observed for large-sized ions (K, Rb, Cs) [16,23,25,47,60]. For the method of synthesis of methylsurrounded Cu-CLMSs, a universal approach that included alkaline hydrolysis [61,62,63] of MeSi(OMe)_3_ assisted by the action of corresponding hydroxide MOH (M = Na, K, Rb, or Cs), was chosen. Then in situ formed alkaline metal siloxanolate [PhSi(O)OM]_x_ species were brought into the exchange interaction with copper(II) chloride (Scheme 1). Characteristically in all cases arising CLMSs **1**–**4** adopted rare for metallasilsesquioxanes octacopper sandwich geometry (CCDC search revealed only two publications reported on the structure of Cu_8_ silsesquioxanes [64,65]). Noteworthy is that the presence of a large inner void in the sandwich structure of complexes reported in [64,65] favors its filling by different encapsulated species, i.e., pyrazine [64], and acetonitrile or sodium acetate [65]. According to these regulations, compounds [(MeSiO_2_)_8_]_2_Cu_8_(DMF)_8_ (EtOH)] • 2.75 (DMF) • 0.25 (Me_2_CO) **1** (isolated in 37% yield), {[(MeSiO_2_)_8_]_2_Cu_8_ (HCOO) (EtOH)_4_ K_2_ (MeCOO)] • 2 (EtOH)}_n_ **2** (31% yield), [(MeSiO_2_)_8_]_2_Cu_8_ (DMF)_8_ Rb (MeO) (EtOH)] **3** (26% yield), {[(MeSiO_2_)_8_]_2_Cu_8_ Br_2_ (DMF)_6_ Cs_2_] • 2 (DMF)_sq_} **4** (42% yield) also feature the encapsulation phenomenon. More precisely, sodium-assisted synthesis (Scheme 1, compound **1**), followed by the addition of dimethylformamide/acetone mixture as a crystallization facilitator, provided mixed-ligated complex **1**. One could see (Figure 1) that while eight DMF molecules coordinate the copper centers of the compound, the ethanol molecule occupies the void of the cage structure. The top view of complex **1** clearly shows the non-regular octagon structure of a prism-like cage. This deviation is also obvious from the comparison of the Cu…Cu distances between opposite copper centers. All four possible Cu…Cu distances are different from each other and they vary in the range from 7.3781(7) Å to 7.6773(7) Å. Two factors potentially responsible for the distortion of the cage could be discussed: (i) the presence of the before mentioned encapsulated ethanol specie (its oxygen center coordinates only two of eight copper sites), and (ii) the variation of the location style of DMF molecules coordinating eight copper centers. Namely, while seven DMF ligands are located in the positions parallel to the basal plane of the copper ions, the eighth DMF molecule is arranged perpendicular to it. Recently, we reported on a similar effect (rotation of acetone solvates [48]) that causes a “shape switch effect” for the hexanuclear copperphenylsilsesquioxanes—from a regular form to an ellipsoid-like one. In the case of complex **1**, due to the presence of the encapsulated component (which was not the case for Ref [48]), we could not state precisely if the distortion of the prismatic geometry of **1** is a consequence of DMFs rotation exclusively. Most probably, both factors are influential and act in parallel.

**Figure 2:**
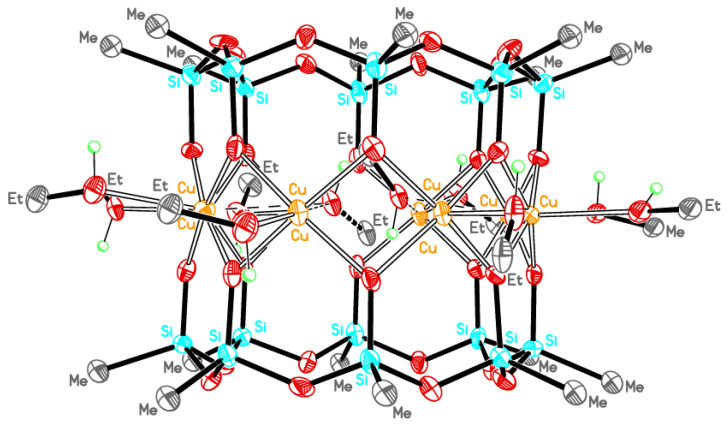

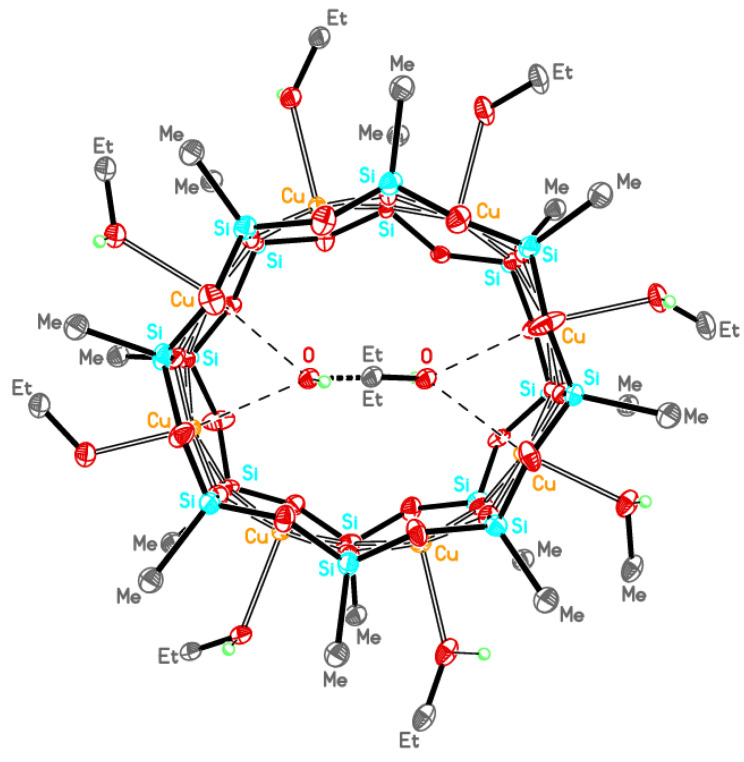
Two projections of molecular structure of complex [(MeSiO_2_)_8_]_2_Cu_8_(EtOH)_7_(MeOH)_0.25_] • EtOH **1a**.

The self-assembly and formation of the Cu_8_-based geometry of CLMS **1** are easily reproduced. Additional runs of synthesis (in an alcohol-only reaction system) smoothly gave complex **1a** [(MeSiO_2_)_8_]_2_Cu_8_(EtOH)_7_(MeOH)_0.25_] • EtOH (Figure 2) in 59% yield. A discussion of the structural features of **1a** is given in Section 3.3.

**Figure 3:**
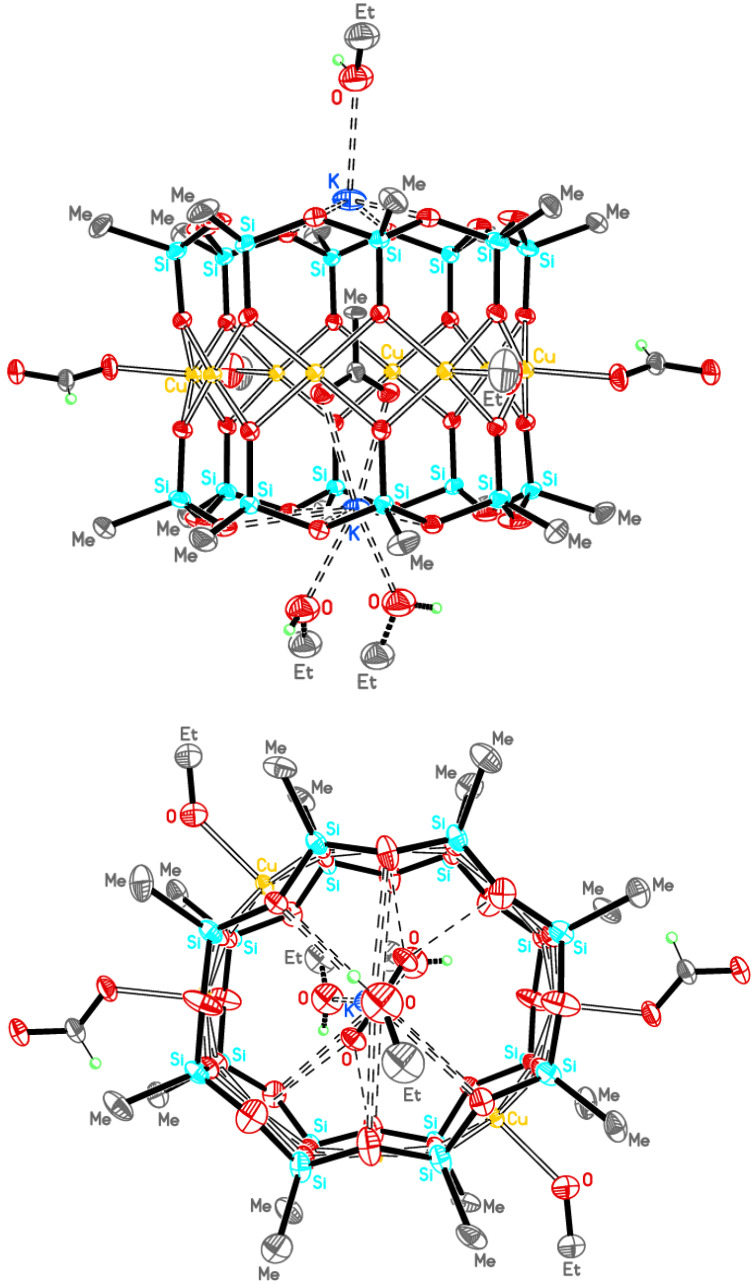
Two projections of molecular structure of complex [(MeSiO_2_)_8_]_2_Cu_8_ (HCOO) (EtOH)_4_ K_2_ (MeCOO)] • 2 (EtOH)}_n_ **2**; the solvate ethanol molecules are not shown.

**Figure 4:**
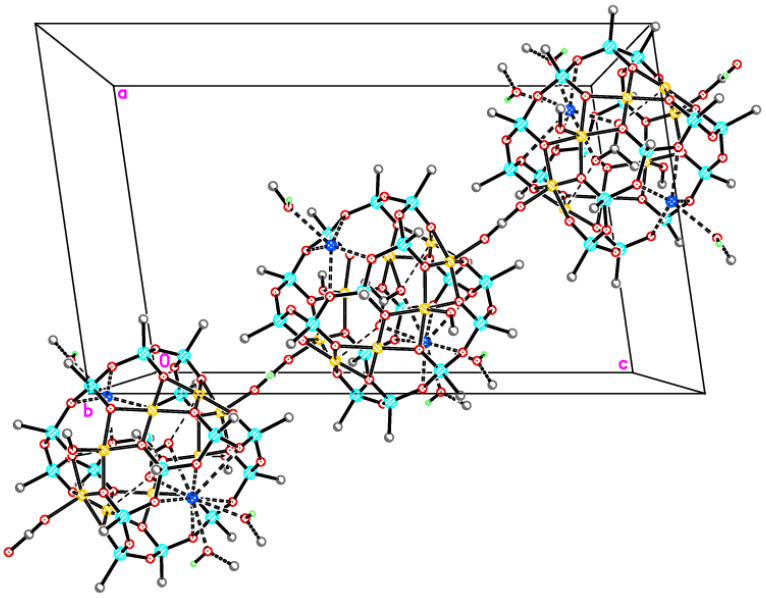
1D coordination polymer structure in structure of **2**; linked via formate bridges.

Next, a similar synthesis as complex **1** has been performed for KOH-involved reaction conditions (Scheme 1). This reaction provided octacopper methylsilsesquioxane complex **2** (Figure 3), whose cage geometry at the first sight is a close analog of compound **1** (Figure 3 top). In point of fact, several deep differences between the two of these compounds could be drawn. First, the shape of the cage of **2** is much ellipsoid in comparison to **1**—Cu…Cu distances in minor and major axes in ellipse fragment are equal to 6.8940(9) Å and 7.9608(7) Å, respectively. The other two opposite Cu…Cu distances are almost equal to each other (7.4115(9) Å and 7.4388(7) Å). Second, compound **2** features the presence of two potassium ions located in crown ether-like positions to siloxane cycles of silsesquioxane ligands. Each potassium center coordinates four oxygen atoms of the corresponding siloxane cycle. It is intriguing that the nature of the anions for potassium cations is different, some of them are carboxylate fragments that appeared as a consequence of the partial oxidation of alcohols present in the synthesis/crystallization system. This is in perfect accord with several earlier reports [49,65,66,67]. Precisely, the first type of anion in **2** is an encapsulated (in axial position) acetate fragment, with acetate oxygen atoms additionally coordinating four of eight copper centers. The next anion type in **2** is an external formate laying in the basal plane of the copper belt of the cage. Additionally, the formate ligand coordinates one of the copper centers. Symptomatically that formate species plays the role of a linker between neighboring cages causing an assembly of 1D coordination polymer (Figure 4). To the best of our knowledge, formate linkers have never been reported as the bridging components for CLMS’ coordination polymers, thus compound **2** shows new possibilities for the supramolecular chemistry of metallasilsesquioxanes. Finally, the last two copper ions in **2** are bonded to ethanol solvates.

**Figure 5:**
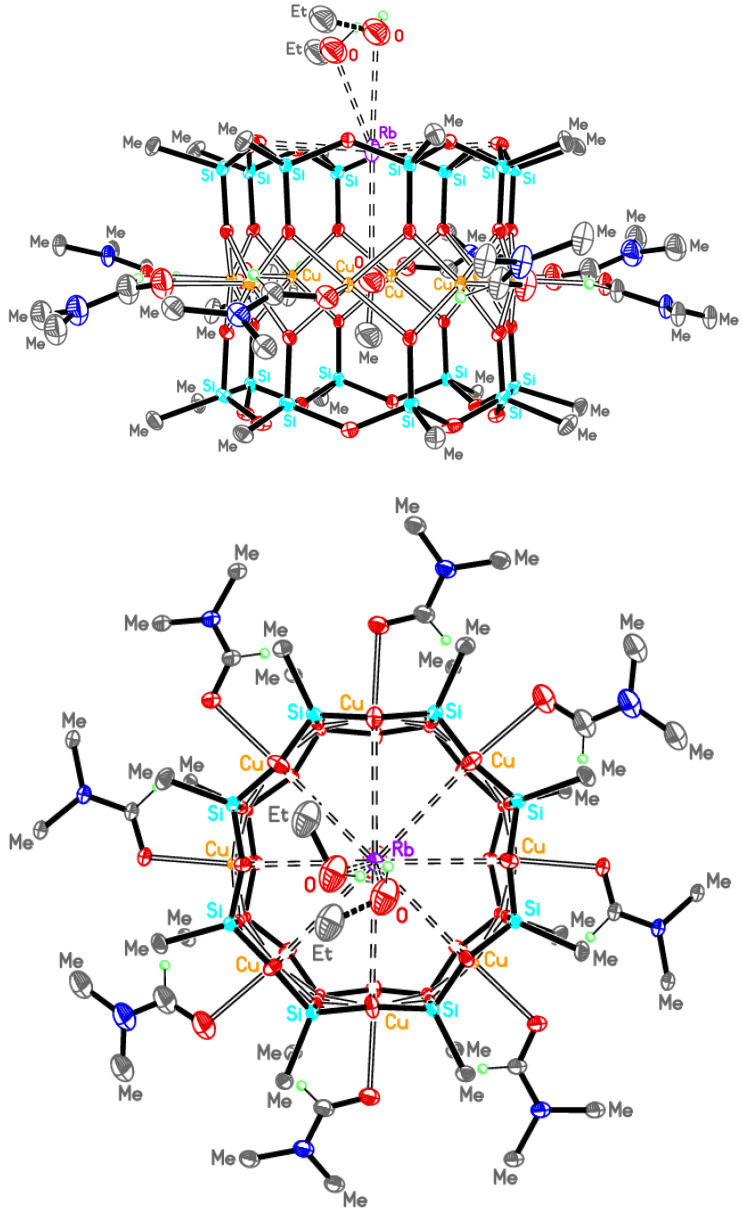
Two projections of molecular structure of complex [(MeSiO_2_)_8_]_2_Cu_8_ (DMF)_8_ Rb (MeO) (EtOH)] **3**.

Then, the shift to RbOH_aq_ as a starting reagent (Scheme 1) allowed isolation of octacopper methylsilsesquioxane complex **3** (Figure 5). Compound **3**, despite the same nuclearity, exhibits significant individuality in comparison to predecessors **1** and **2**. Due to the larger size of the ion, rubidium center in **3** coordinates all eight oxygen atoms in the siloxane cycle of the silsesquioxane ligand. This causes the formation of a more regular prismatic type of cage in comparison to **2**. The range between the shortest (7.3674(1) Å) and the longest (7.6497(9) Å) opposite Cu…Cu distance is just ~0.28 Å (for the ellipsoid cage of **2**—~1.07 Å). The role of counterion for rubidium cation in complex **3** is played by the OMe group (located in the axial position). The oxygen atom of the methoxy group is laying in a basal plane of the copper belt and does not coordinate with any of the copper centers. In turn, each copper ion is ligated by an external DMF molecule. The screening effect of DMF solvates prevents the assembly of any supramolecular derivatives of **3** via its copper centers. The same could be said regarding screening ethanol solvate and coordinating rubidium ions. This also prevents the formation of the coordination polymer structure despite the significant coordinative potential of rubidium centers towards siloxanes, which could be fruitful in supramolecular assembly [25,68,69,70]. 

**Figure 6:**
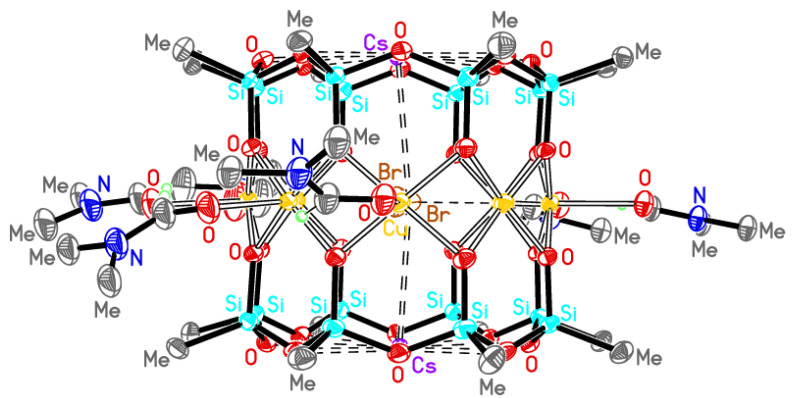

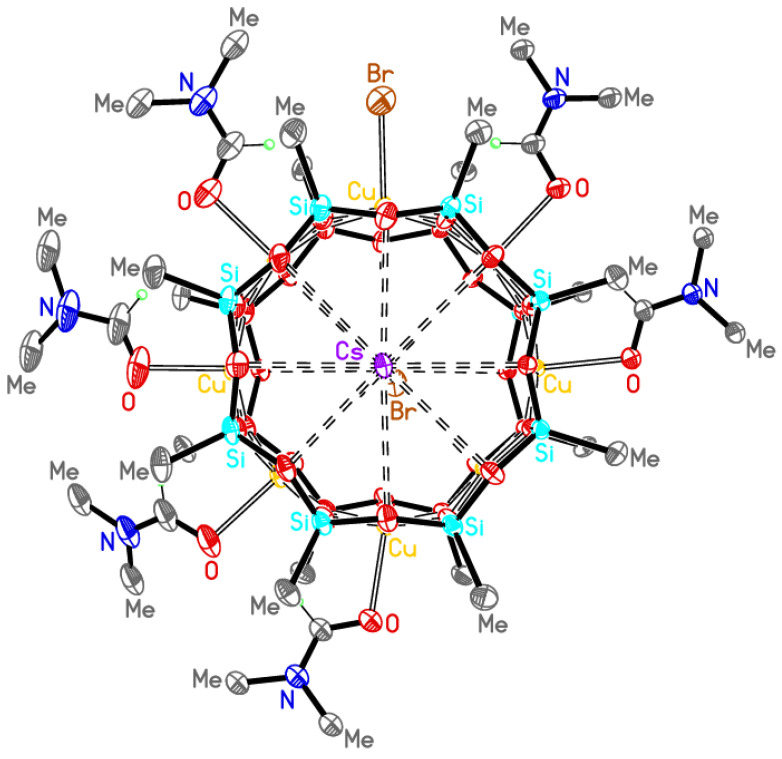
Two projections of molecular structure of complex [(MeSiO_2_)_8_]_2_Cu_8_ Br_2_ (DMF)_6_ Cs_2_] • 2 (DMF)_sq_ **4**.; the solvate DMF molecules are not shown.

**Figure 7:**
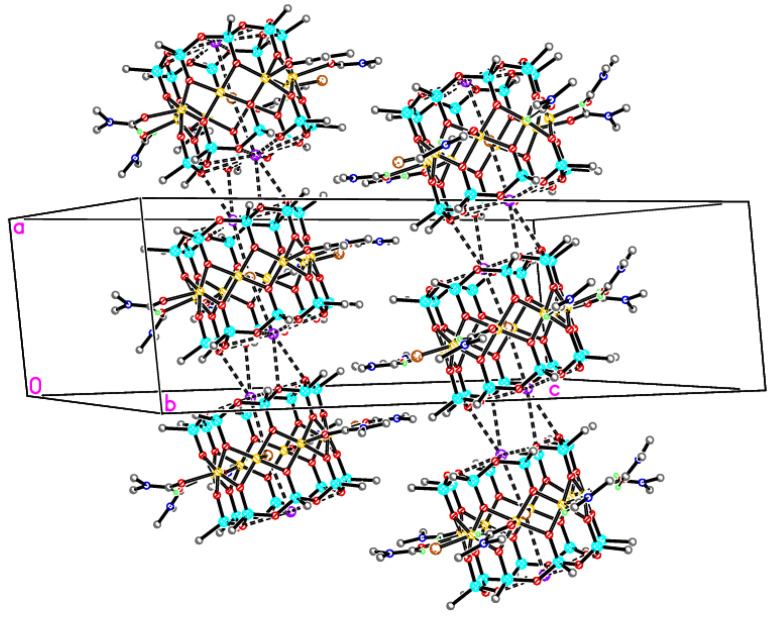
1D coordination polymer structure of **4** via crown ether contacts of cesium and oxygen atoms of neighboring cages in crystal packing.

Finally, the shift to CsOH_aq_ as a starting reagent (Scheme 1) allowed isolation of octacopper methylsilsesquioxane complex **4** (Figure 6). It is worthwhile to notice that we failed to crystallize the compound from general reaction conditions. Keeping in mind the recently reported high efficiency of exchange reactions with Et_4_NCl (or Ph_4_PCl) for the isolation of Ln-based CLMSs [14,20,22], we decided to use a similar reaction with Me_4_NBr here. To our surprise, instead of the formation of “cation-involved CLMS” (as in the case of Et_4_N^+^/Ph_4_P^+^ based compounds in Refs [14,20,22]), compound **4** is “anion-involved” CLMS bearing two bromide groups. It is intriguing that bromides are completely different in their role in the composition of **4**. Namely, when the first of them is located in an external position to the cage and connected to copper ion, the second bromide is encapsulated into the inner void of the cage. The presence of additional bromide as carriers of two negative charges requires charge-balancers. These latter (cesium ions) are found in crown ether positions by siloxane cycles of silsesquioxane ligands and bring a similar effect as the rubidium ion in complex **3**. Namely, cesium centers, owing to their large ion size, coordinate all eight oxygen centers in the siloxane cycle. Keeping this fact in mind, as well as the presence of DMF molecules as the only solvates in the composition of **4**, one could expect a regular shape type of cage fragment of **4**. In fact, the difference between the shortest (7.2469(2) Å) and the longest (7.7244(2) Å) distances between opposite copper ions is significantly larger than the before mentioned value for **3**—~0.48 Å. This points to the irregularity of structure **4**. Indeed, the presence of one Cu-Br fragment and one copper center without any solvate surrounding shows the inequality of copper sites. It is important to note that the copper atom free from an external ligand is linked to the encapsulated bromide anion at a distance of 3.069(3) Å. Each of the remaining six copper ions is ligated by a DMF molecule. This is especially intriguing because an absence of any screening solvates at cesium centers allows the assembly of an unusual supramolecular structure of **4**. 1D coordination polymer **4** (Figure 7) is realized due to mutual Cs…O contacts between the cesium ion of one cage and the oxygen atom from the siloxane cycle of the neighboring cage. Thus, the rectangular linking unit Cs_2_O_2_ is connected to two cages. Symptomatically, this unit is repeated in a diagonal manner when the next cage fragment joins, giving non-linear supramolecular geometry. 

Remarkably, the detailed structural analysis of the complexes studied here allows revealing the characteristic criterion of the template effect of encapsulated anions/molecules, namely, the copper atoms bound to the template anions/molecules remain externally “naked” (see complexes **2** and **4**). The “naked” environment of the copper(II) cations is absolutely unusual taking into account the favorable [4+2]-coordination geometry for it due to the Jahn–Teller effect. Consequently, in contrast to the acetate anion in **2** and bromide anion in **4**, the encapsulated ethanol molecule in **1** and the methoxy anion in **3** are not template agents. This statement is in perfect agreement with the Cu_8_- and Cu_9_-cage-like complexes studied by us previously (with the template agents of the acetate anion and DMSO molecule, respectively) [65,66].

### 2.2. Catalytic Studies

**Figure 8:**
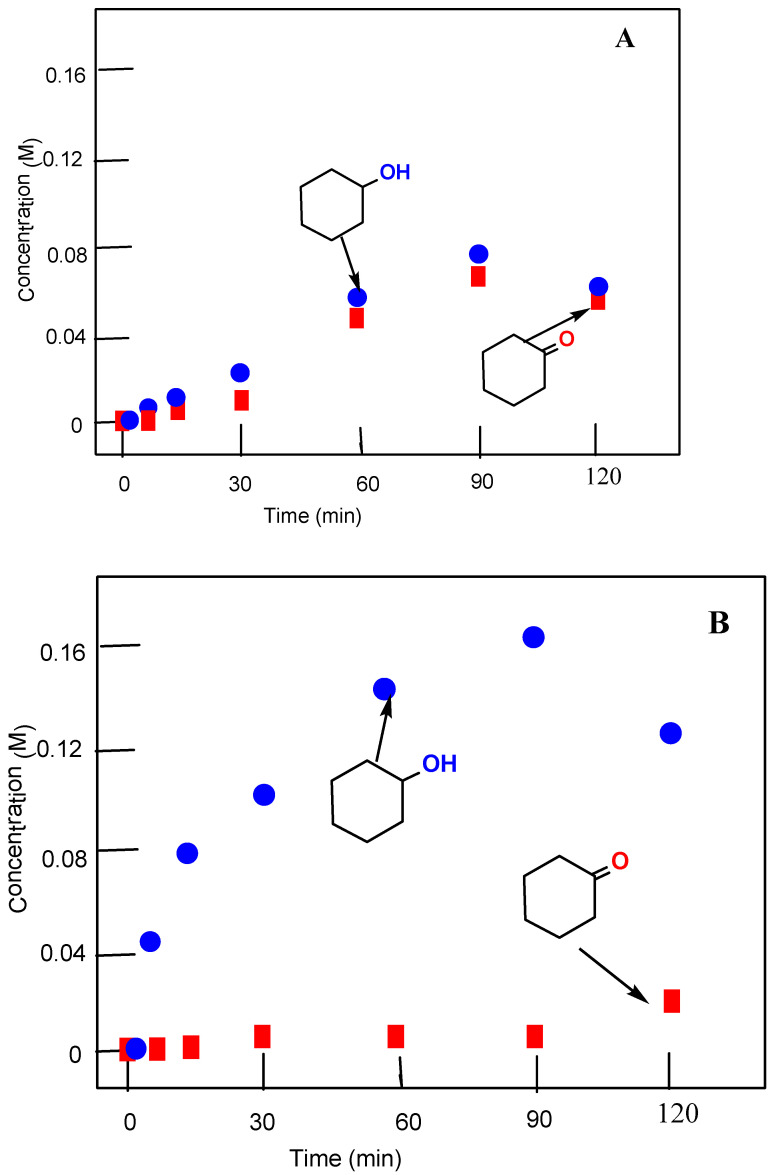
Accumulation of cyclohexanol and cyclohexanone in oxidation of cyclohexane (0.46 M) with hydrogen peroxide (2.0 M, 50% aqueous) catalyzed by compound **4** (5 × 10^−4^ M) in the presence of HNO_3_ (0.05 M) in MeCN at 50 °C. Concentrations of cyclohexanone and cyclohexanol were determined before reduction of the PPh_3_ (Graph **A**), and the same reaction after reduction of the aliquots with solid PPh_3_ (Graph **B**).

**Figure 9:**
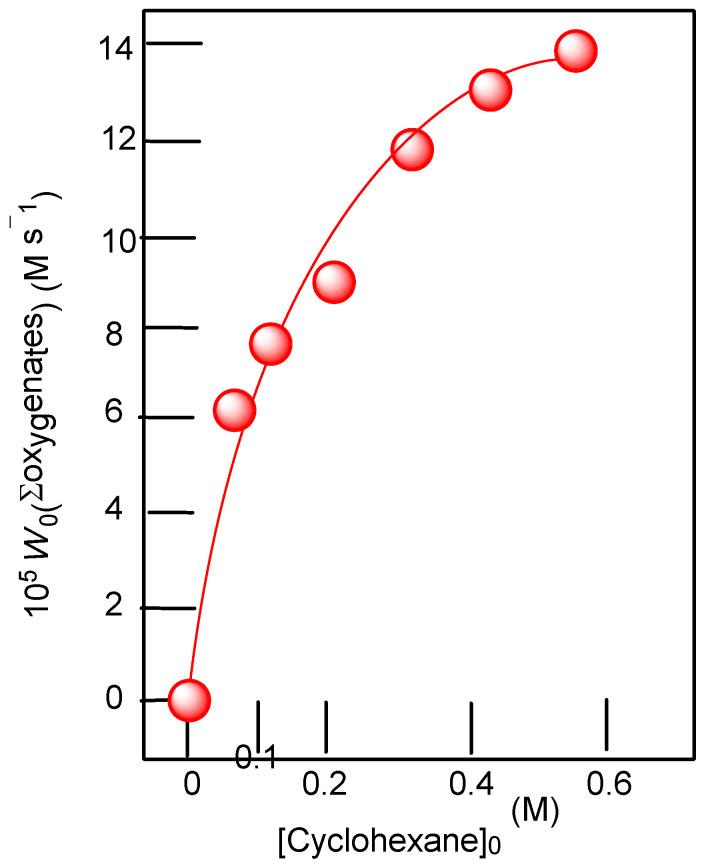
Dependence of the initial rate of oxygenate (sum cyclohexanol + cyclohexanone) formation *W*_0_ for complex **4** on the initial concentration of cyclohexane (Initial concentrations: Catalyst 5 × 10^−4^ M, H_2_O_2_ 1.5 M, HNO_3_ 0.05 M, CH_3_CN up to 2.5 mL, T = 50 °C).

**Figure 10:**
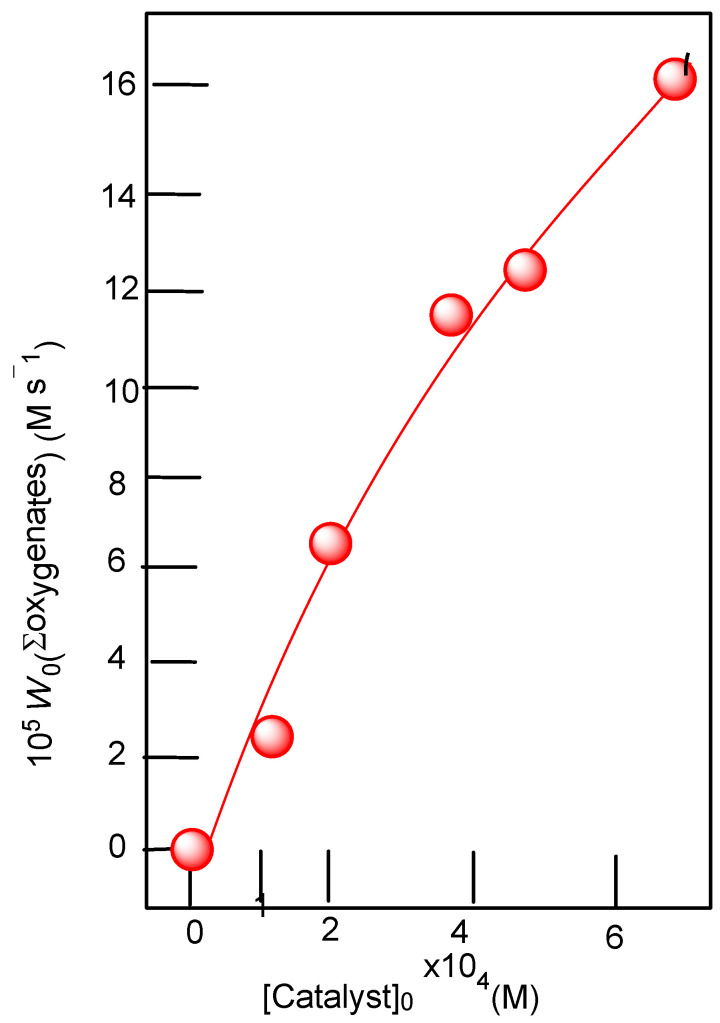
Dependence of the initial rate of oxygenate (sum cyclohexanol + cyclohexanone) formation *W*_0_ for complex **4** on the initial concentration of catalyst. (Initial concentrations: cyclohexane 0.46 M, H_2_O_2_ 1.5 M, catalyst 5 × 10^−4^ M, HNO_3_ 0.05 M, CH_3_CN up to 2.5 mL, T = 50 °C).

**Figure 11:**
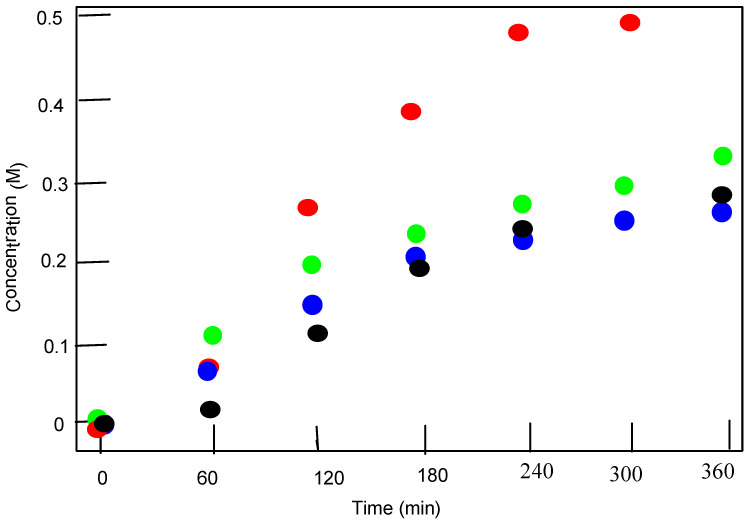
Accumulation of cyclohexanone (yield = 60%, TON = 600) in oxidation of cyclohexanol (0.5 M, green—curve 1), acetophenone (yield = 98%, TON = 980) in oxidation of phenylethanol (0.5 M, red—curve 2), 2-heptanone (yield = 48%, TON = 480) in oxidation of 2-heptanol (0.5 M, blue—curve 3) and 2-hexanone (yield = 50%, TON = 500) in oxidation of 2-hexanol (0.5 M, black—curve 4) with *tert-*butyl hydroperoxide (1.5 M, 70% aqueous) catalyzed by compound **4** (5 × 10^−4^ M) in MeCN at 50 °C.

We discovered that complex **4** exhibits high catalytic activity in the oxidation of saturated hydrocarbons. Accumulations of the products are presented in Figure 8, Figure 9, Figure 10 and Figure 11. Oxidation of cyclohexane with hydrogen peroxide according to gas chromatography results in the formation of a mixture of cyclohexanol and cyclohexanone. Adding triphenylphosphine to the reaction mixture according to the method proposed previously by one of us [71,72], leads to a sharp increase in the concentration of cyclohexanol compared to the concentration of cyclohexanone, as shown in Figure 8B. (The ratio of cyclohexanone to cyclohexanol after adding triphenylphosphine, for example, is about 0.04 (0.007 M/0.142 M after 60 min). At the same time in the absence of phosphine, the ratio of cyclohexanone to cyclohexanol for complex **4** is about 0.7 (0.042 M/0.059 M after 60 min) Figure 8A. The effect of adding triphenylphosphine to a sample of the reaction mixture can be explained by the fact that the main product of the oxidation reaction is alkyl hydroperoxide. In the absence of phosphine, it decomposes to form a ketone and alcohol in comparable amounts in an injector of GC. Triphenylphosphine reduces an alkyl hydroperoxide to alcohol, so we see a sharp increase in the amount of alcohol on the chromatogram. The total yield of alcohol and ketone in the oxidation of cyclohexane catalyzed by complex **4** after 1.5 h is 35%, and TON is 320. To carry out the oxidation reaction of alkanes, it is necessary to add nitric acid. When carrying out reactions in the absence of nitric acid, only the decomposition of hydrogen peroxide (catalase activity) is observed and the yield of oxidation products is not more than 0.5% in two hours. In this case, rapid precipitation of a brown precipitate is observed, apparently, the decomposition of the complex occurs.

We investigated the oxidation of n-heptane with hydrogen peroxide catalyzed by complex **4**. The results are given below (initial concentrations: n-heptane 0.5 M, H_2_O_2_ 1.5 M, catalyst 5 × 10^−4^ M, HNO_3_ 0.05 M, CH_3_CN up to 2.5 mL). After two hours, the following concentrations of (M) alcohol isomers were obtained (after reduction with triphenylphosphine maximum yield of products in the oxidation of n-heptane 10% was obtained): C (1) 0.0003M; C (2) 0.006; C (3) 0.006M: C (4) 0.003M. These values give a series of selectivity: C (1):C (2):C (3):C (4) = 1.0:5.6:5.6:5.6. The selectivity parameters for the oxidation of methylcyclohexane were obtained also: 1°:2°:3° = 1.0:4.8:14.2 (after reduction with triphenylphosphine maximum yield of products in the oxidation of methylcyclohexane 12% was obtained). However, the selectivity parameters obtained for n-heptane are slightly higher than the typical selectivity parameters for the oxidation of n-heptane with the participation of hydroxyl radicals [73,74,75]. We see two possible reasons for these differences. First, it is possible that, along with the hydroxyl radical, another oxidizing species is generated in the system, which exhibits greater regioselectivity than OH. This can be, in particular, the Cu(III) ion [76]. Secondly, it is possible that the differences in regioselectivity and reactivity are associated with steric hindrances in the access of an alkane to the reaction center near which hydroxyl radicals are generated. Consideration of the regio-and bond-selectivity in the oxidation of n-heptane and methylcyclohexane and the dependences of initial rates of oxygenates formation (Figure 9 and Figure 10) indicates that the oxidation proceeds in the main with the participation of free hydroxyl radicals.

### 2.3. Oxidation of Alcohols 

Aliphatic and aromatic alcohols are oxidized with hydrogen peroxide with very low efficiency, however to the use of *tert*-butyl hydroperoxide, they give ketones as oxidation products in good to high yields. It should be noted that these reactions do not require the addition of nitric acid. The accumulation of products in such reactions is shown in Figure 11. We would like to mention that the principle of catalytic activity of complex **4** is a precatalytic one. In homogeneous conditions (and in the presence of peroxide and acid cocatalyst) complex **4** modifies into catalytic active species, which further provide efficient oxidations. The advantage of metallasilsesquioxane precatalysts is a high activity of catalysis; these cage catalysts show superior activity in comparison with the simple copper salts and complexes [77].

## 3. General Experimental Considerations

**Figure 12:**
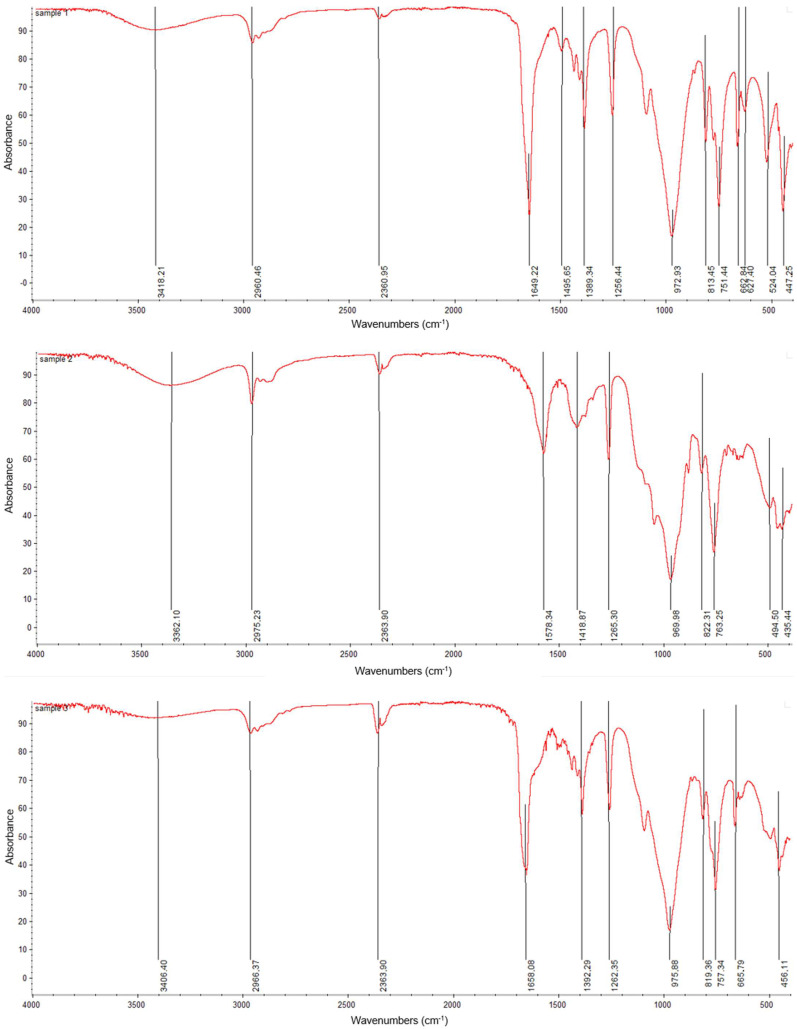

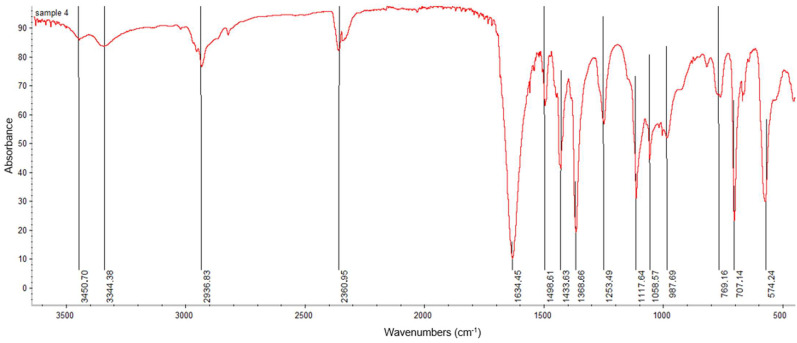
From top to bottom. IR spectra for **1**–**4**.

**Figure 13:**
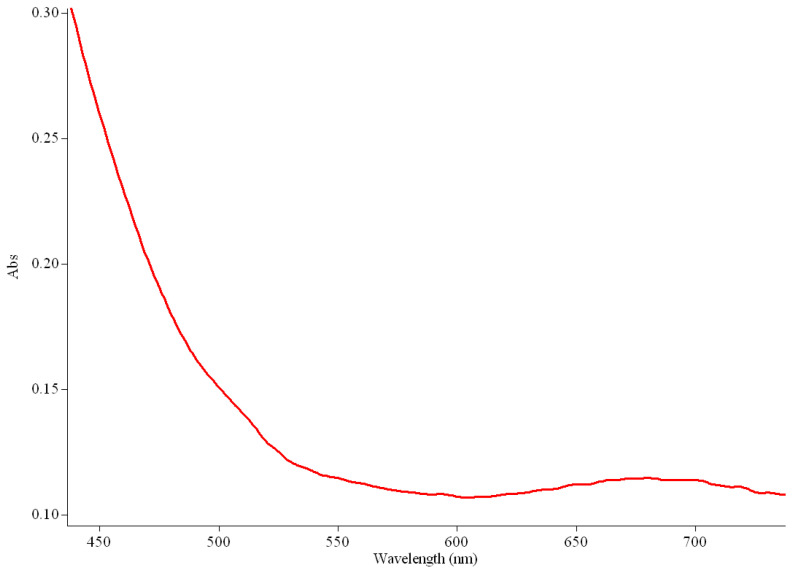
UV-vis spectrum for **4**.

All reagents were purchased from the usual suppliers (Sigma, Fluka) and used without further purification. RbOH and CsOH were used as 50% w/w water solutions. Elemental analyses were carried out with an XRF spectrometer VRA-30 and Eurovector EA-3000 analyzer. IR spectra of the compounds (KBr pellets, Figure 12, see below) were measured on a Shimadzu IR Prestige 21 FT-IR Spectrophotometer equipped with an MCT detector using a Miracle single reflection ATR unit by Pike. Set of signals: 1650–1550 cm^−1^ (νC=N), 1420–1380 cm^−1^ (ν_stretch_C–Si), 1120 cm^−1^ (ν_as_Si–O–Si), 1050 cm^−1^ (ν_s_Si–O–Si), 960 cm^−1^ (ν_as_Si–O in Si–O–M fragment), 820 cm^−1^ and 760 cm^−1^ (ν_wag_C–Si). UV-Vis spectra (10 mm optical path length, acetone solutions) were recorded on a Cary 50 spectrophotometer. Figure 13 is to exemplify a general view of spectra (for complex **4**, see below).

### 3.1. Syntheses of ***1***–***4***

In a typical procedure, 1.5 g (11 mmol) of MeSi(OMe_3_)_3_ and 11.7 mmol of corresponding MOH (M = Na, K, Rb, or Cs, see below for details) were heated at reflux in 40 mL of ethanol for 1.5 h. Then, 0.73 g (5.43 mmol) of CuCl_2_ was added and the resulting mixture was heated at reflux for 3 h. Then the solution was stirred without heating for 24 h, followed by centrifugation of the precipitate.

For compound [(MeSiO_2_)_8_]_2_Cu_8_(DMF)_8_ (EtOH)] • 2.75 (DMF) • 0.25 (Me_2_CO) **1**: Mass of MOH (NaOH) = 0.47 g. Crystallization of filtrate (after the addition of 20 mL of DMF/acetone (1:1, *v*:*v*) gave a crystalline material in a week, including single crystals that were used for X-ray diffraction and elemental analyses. Anal. Calcd for [(CH_3_SiO_2_)_8_]_2_Cu_8_(C_3_H_7_NO)_10.25_(C_2_H_5_OH)(C_3_H_6_O)_0.25_: C, 23.59; H, 5.09; Cu, 20.17; N, 5.70; Si, 17.83. Found: C, 22.14; H, 4.91; Cu, 20.29; N, 5.61; Si, 17.91. The rest of crystalline material was dried in vacuum. Yield: 0.28 g (37%).

For compound [(MeSiO_2_)_8_]_2_Cu_8_(EtOH)_7_(MeOH)_0.25_] • EtOH **1a**: Mass of MOH (NaOH) = 0.47 g. Crystallization of filtrate (after the addition of 10 mL of methanol) gave a crystalline material in 3–4 days, including single crystals that were used for X-ray diffraction and elemental analyses. Anal. Calcd for [(CH_3_SiO_2_)_8_]_2_Cu_8_(CH_3_OH)_0.25_(C_2_H_5_OH)_8_: C, 18.56; H, 4.69; Cu, 24.36; Si, 21.53. Found: C, 18.41; H, 4.58; Cu, 24.50; O, 30.97; Si, 21.60. The rest of crystalline material was dried in vacuum. Yield: 0.45 g (59%).

For compound {[(MeSiO_2_)_8_]_2_Cu_8_(HCOO)(EtOH)_4_K_2_(MeCOO)] • 2 (EtOH)}_n_ **2**: Mass of MOH (KOH) = 0.66 g. Crystallization of filtrate gave a crystalline material in a week, including single crystals that were used for X-ray diffraction and elemental analyses. Anal. Calcd for [(CH_3_SiO_2_)_8_]_2_Cu_8_(HCOO)(C_2_H_5_OH)_6_K_2_(CH_3_COO): C, 17.17; H, 4.09; Cu, 23.44; K, 3.61; Si, 20.72. Found: C, 17.01; H, 3.95; Cu, 23.53; K, 3.69; Si, 20.81. The rest of crystalline material was dried in vacuum. Yield: 0.35 g (31%).

For compound [(MeSiO_2_)_8_]_2_Cu_8_ (DMF)_8_ Rb (MeO) (EtOH)] **3**: Mass of MOH (RbOH) = 1.20 g. Crystallization of filtrate (after the addition of 15 mL of DMF) gave a crystalline material in a week, including single crystals that were used for X-ray diffraction and elemental analyses. Anal. Calcd for [(CH_3_SiO_2_)_8_]_2_Cu_8_(C_3_H_7_NO)_8_(C_2_H_5_OH)Rb(CH_3_O): C, 21.01; H, 4.63; Cu, 20.69; N, 4.56; Rb, 3.48; Si, 18.28. Found: C, 20.69; H, 4.50; Cu, 20.77; N, 4.41; Rb, 3.59; Si, 18.37. The rest of crystalline material was dried in vacuum. Yield: 0.28 g (26%).

For compound {[(MeSiO_2_)_8_]_2_Cu_8_ Br_2_ (DMF)_6_ Cs_2_] • 2 (DMF)_sq_} **4**: Mass of MOH (CsOH) = 1.75 g. Crystallization of filtrate was found unsuccessful and additional exchange reaction with Me_4_NBr (0.42 g, 2.75 mmol) was performed. Mixture was stirred without heating for 24 h, followed by centrifugation of precipitate. Resulting filtrate (after the addition of 15 mL of DMF) gave a crystalline material in a week, including single crystals that were used for X-ray diffraction analysis. Anal. Calcd for [(CH_3_SiO_2_)_8_]_2_Cu_8_(C_3_H_7_NO)_8_Cs_2_Br_2_: C, 17.66; H, 3.85; Br, 5.87; Cs, 9.77; Cu, 18.69; N, 4.12; Si, 16.52. Found: C, 17.52; H, 3.79; Br, 5.92; Cs, 9.82; Cu, 18.77; N, 4.05; Si, 16.60. The rest of crystalline material was dried in vacuum. Yield: 0.54 g (42%).

### 3.2. X-ray Crystal Structure Determination

The single-crystal X-ray diffraction study of **1–4** was carried out on a four-circle Rigaku Synergy S diffractometer equipped with a HyPix6000HE area-detector (*T* = 100 K, graphite monochromator, shutterless *ω*-scanning mode). The data were integrated and corrected for absorption by the *CrysAlisPro* program [78]. The single-crystal X-ray diffraction study of **1a** was carried out on the ‘RSA’ beamline (*T* = 100 K, *λ* = 0.75270 Å) of the Kurchatov Synchrotron Radiation Source using a Rayonix SX165 detector. In total, 720 frames were collected with an oscillation range of 1.0° in the *φ* scanning mode using two different orientations for the crystal. The semi-empirical correction for absorption was applied using the *Scala* program [79]. The data were indexed and integrated using the utility *iMOSFLM* from the CCP4 software suite [80,81]. For details, see Table 1.

The structures were solved by intrinsic phasing modification of direct methods [82], and refined by a full-matrix least-squares technique on *F*^2^ with anisotropic displacement parameters for all non-hydrogen atoms. In the case of **4**, all attempts to model and refine positions of the solvate DMF molecules were unsuccessful. Therefore, their contribution to the total scattering pattern was removed by use of the utility *SQUEEZE* in PLATON15 [83]. The hydrogen atoms of the hydroxyl groups were objectively localized in the difference-Fourier maps and included in the refinement within the riding model with fixed isotropic displacement parameters [*U*_iso_(H) = 1.5*U*_eq_(O)]. The other hydrogen atoms were placed in calculated positions and refined within the riding model with fixed isotropic displacement parameters [*U*_iso_(H) = 1.5*U*_eq_(C) for the CH_3_-groups, and 1.2*U*_eq_(C) for the other groups]. All calculations were carried out using the *SHELXTL* program [84,85].

Crystallographic data have been deposited with the Cambridge Crystallographic Data Center, CCDC 2232957 (**1**), CCDC 2235882 (**1a**), 2232958 (**2**), 2232959 (**3**), and 2232960 (**4**). Copies of this information may be obtained free of charge from the Director, CCDC, 12 Union Road, Cambridge CB2 1EZ, UK (fax: +44 1223 336033; e-mail: deposit@ccdc.cam.ac.uk or www.ccdc.cam.ac.uk).

### 3.3. Structural Features of Complex ***1a***

Similarly to complex **1**, the inner void of cage structure **1a** is occupied by ethanol molecules. The rest of the alcohol-solvating ligands (seven ethanol and one methanol) occupy external positions at the basal plane of complex **1a** and coordinate copper centers. These differences in the nature of solvating ligands cause some transformations in non-regular octagon cages **1** and **1a**. For example, distances between opposite copper centers in **1** vary between 7.3781(7) Å and 7.6773(7) Å. In turn, for compound **1a** this deviation is larger (between 7.3789(3) Å and 7.8144(3) Å).

### 3.4. Catalytic Studies

All reactions were carried out in air in thermostatically controlled cylindrical glass vessels with vigorous stirring. The total volume of the reaction solution was 5 mL (WARNING: the combination of air or molecular oxygen and H_2_O_2_ with organic compounds can be explosive at elevated temperatures!). Initially, a portion of a 50% aqueous solution of hydrogen peroxide or tert-butyl hydroperoxide (70%) (for alcohols) was added to a solution of the catalyst (added in dry form), HNO_3_ (from the stock solution) and the substrate in acetonitrile). Attribution of peaks was made by comparison with chromatograms of authentic samples). Usually, samples were analyzed twice, i.e., before and after the addition portion by portion of the excess of solid PPh_3_. Aliquots of the reaction solution were analyzed by GC (CH3NO2 (250 μL) was used as an internal standard) (the instrument chromatograph—Cromas, FFAP/OV-101 20/80 *w*/*w* fused silica capillary column, 30 m × 0.2 mm × 0.3μm, argon as a carrier gas). The reactions of alkanes and alcohols were stopped by cooling and were usually analyzed twice, i.e., before and after addition of excess solid PPh_3_.

## 4. Conclusions

A series of octacopper cage methylsilsesquioxanes **1**–**4** were prepared and characterized. The strong influence of steric factors (nature of the substituent at silicon atom) on the self-assembly of coppersilsesquioxanes was found, keeping in mind that the archetype structure of phenylsilsesquioxane derivatives is a hexacopper cage. A change of geometry of prismatic cages **1**–**4** caused by the variation of their composition (different alkaline metal ions, solvates, or encapsulated species) was detected. It revealed the characteristic criterion of the template effect of encapsulated anions/molecules for the cage-like copper complexes. Noteworthy is that the large size of the inner void of octacopper silsesquioxanes points to the significant potential of further studies of high nuclear metallasilsesquioxanes in the context of potentially intriguing encapsulation effects. The same concerns observed in different types of intercage connectivity provided 1D coordination polymers for compound **2** (formate linkers) and **4** (crown ether contacts). Noteworthy is that carboxylate bridges in structures of cage metallasilsesquioxane coordination polymers were never reported before this work. This points to many prospects for supramolecular chemistry involving metallasilsesquioxanes. Complex **4** exhibits high catalytic activity in the oxidation of alkanes (cyclohexane, *n*-heptane, and methylcyclohexane) with hydrogen peroxide and alcohols (phenylethanol, cyclohexanol, 2-heptanol, and 2-hexanol) with *tert*-butyl hydroperoxide. Measurement of *n*-heptane regioselectivity and bond selectivity in the reactions of methylcyclohexane with H_2_O_2_ allows us to conclude that hydroxyl radicals play an important role in these oxidation reactions. It is possible that, along with the hydroxyl radical, another oxidizing species (it may be Cu(III)-ion) is generated in the system. The starting products in reactions with alkanes are alkyl hydroperoxides, which are reduced to the corresponding alcohols with an excess of triphenylphosphine (PPh_3_).

## Figures and Tables

**Table 1:** Crystal Data and Structure Refinement for Compounds **1–4**.

Identification Code	1 • 2¾C_3_H_7_NO • ¼C_3_H_6_O	1a	2 • 2C_2_H_6_O	3	4 • 2C_3_H_7_NO
Empirical formula	C_51_H_130.75_Cu_8_N_10.75_O_44_Si_16_	C_129_H_388_Cu_32_O_161_Si_64_	C_31_H_88_Cu_8_K_2_O_42_Si_16_	C_43_H_113_Cu_8_N_8_O_42_RbSi_16_	C_34_H_90_Br_2_Cs_2_Cu_8_N_6_O_38_Si_16_
Formula weight	2556.76	8347.75	2169.06	2457.73	2574.58
Temperature, K	100(2)	100(2)	100(2)	100(2)	100(2)
Crystal size, mm	0.20 × 0.18 × 0.15	0.12 × 0.06 × 0.03	0.23 × 0.13 × 0.12	0.15 × 0.13 × 0.12	0.35 × 0.05 × 0.03
Wavelength, Å	0.71073	0.75270	1.54184	1.54184	1.54184
Crystal system	Monoclinic	Orthorhombic	Monoclinic	Monoclinic	Monoclinic
Space group	*P*2_1_/*c*	*P*2_1_2_1_2	*C*2/*c*	*P*2_1_/*n*	*P*2_1_/*c*
*a*, Å	18.7364(2)	28.231(3)	14.60230(14)	17.15942(11)	10.68626(9)
*b*, Å	19.9150(3)	16.8711(14)	22.9138(2)	14.19682(8)	25.0419(2)
*c*, Å	29.0275(3)	17.3129(15)	24.0571(2)	21.78734(10)	35.8364(3)
*α*, deg.	90	90	90	90	90
*β* deg.	94.2936(11)	90	98.2831(9)	90.7073(5)	95.0906(9)
*γ*, deg.	90	90	90	90	90
*V*, Å^3^	10,800.8(2)	8245.9(12)	7965.40(12)	5307.19(5)	9552.15(14)
*Z*	4	1	4	2	4
Density (calc.), g/cm^3^	1.572	1.681	1.809	1.538	1.790
*μ*, mm^−1^	1.804	2.729	6.312	4.679	11.331
*F*(000)	5280	4274	4416	2512	5104
Theta range, deg.	1.996–35.458	1.992–30.992	3.713–77.656	3.716–79.599	3.741–78.402
Index ranges	−28 ≤ h ≤ 29, −31 ≤ k ≤ 26, −44 ≤ l ≤ 46	−38 ≤ h ≤ 38, −22 ≤ k ≤ 22, −23 ≤ l ≤ 23	−18 ≤ h ≤ 18, −26 ≤ k ≤ 28, −30 ≤ l ≤ 30	−21 ≤ h ≤ 21, −18 ≤ k ≤ 18, −21 ≤ l ≤ 27	−13 ≤ h ≤ 13, −31 ≤ k ≤ 31, −45 ≤ l ≤ 45
Reflections collected	215,764	85,332	47,380	67,623	346,984
Independent reflections	44,011 (R_int_ = 0.0482)	21,823 (R_int_ = 0.0701)	8327 (R_int_ = 0.0436)	11,268 (R_int_ = 0.0408)	20,299 (R_int_ = 0.1134)
Reflections observed	29,709	13,993	7365	10,617	18,431
Restraints/parameters	111/1170	71/756	15/462	67/496	0/840
*R*_1_/w*R*_2_ (*I* > 2σ(*I*))	0.0510/0.1203	0.0721/0.1649	0.0556/0.1520	0.0715/0.1463	0.1054/0.2197
*R*_1_/w*R*_2_ (all data)	0.0927/0.1431	0.1124/0.1899	0.0610/0.1594	0.0740/0.1476	0.1116/0.2232
Goodness-of-fit on *F*^2^	1.037	1.022	1.024	1.005	1.028
Extinction coefficient	─	0.00067(6)	─	0.000250(16)	0.000418(18)
*T*_min_/*T*_max_	0.663/0.707	0.716/0.903	0.234/0.500	0.447/0.560	0.090/0.699
Δρ_max_/Δρ_min_, e^.^Å^−3^	1.653/−1.363	2.043/−1.266	1.297/−0.814	2.604/−1.898	2.120/−2.670

## Data Availability

The data presented in this study are available in the article.

## Sample Availability

Samples of the compounds are not available from the authors.
